# RETRACTED ARTICLE: Net Fluorescein Flux Across Corneal Endothelium Suggests Fluid Transport is Driven by Electroosmosis

**DOI:** 10.1007/s00232-015-9849-y

**Published:** 2015-09-30

**Authors:** V. Cacace, C. F. Kusnier, J. Fischbarg

**Affiliations:** ININCA, Conicet, Marcelo T. de Alvear 2270, CP 1122AAJ Buenos Aires, Argentina

This article has been retracted at the request of the Corresponding Author. The retraction has been agreed due to flaws in the data. The authors regret the oversight.

## Electronic supplementary material

Below is the link to the electronic supplementary material.

Supplementary material 1 (mcd 96 kb)

Former article version (pdf 700 kb)

