# Supplementary material for: RETRACTED ARTICLE: Net Fluorescein Flux Across Corneal Endothelium Suggests Fluid Transport is Driven by Electroosmosis
Source: J Membr Biol. 2015 Sep 30;249:197. doi: 10.1007/s00232-015-9849-y (PMC4851691; doi:10.1007/s00232-015-9849-y)
Supplement: Supplementary file 2 — Former article version (pdf 700 kb) [file 232_2015_9849_MOESM2_ESM.pdf]

J Membrane Biol
DOI 10.1007/s00232-015-9849-y

CrossMark

BRIEF COMMUNICATION

# Net Fluorescein Flux Across Corneal Endothelium Suggests Fluid Transport is Driven by Electroosmosis

**V. Cacace[1] · C. F. Kusnier[1] · J. Fischbarg[1]**

RETRACTED ARTICLE

Received: 25 June 2015 / Accepted: 18 September 2015
© The Author(s) 2015. This article is published with open access at Springerlink.com

**Abstract** There is evidence that endothelial fluid transport results from paracellular electroosmosis. If so, it ought to drag solutes along the paracellular route. We have used fluorescein-$Na_2$ as label to investigate this. SV-40-transformed human cultured endothelial cells (J. Bednarz's line) were grown to confluence on permeable membrane inserts held in a $CO_2$ incubator for 1 h, after which the unlabelled side was collected. The fluorescein counts were read for 60 s. The cells are known to transport fluid from the basolateral to the apical side. We determined fluxes for 20 1-h periods (four inserts). We found a statistically significant net flux of fluorescein from the basolateral to the apical side (flux ratio: [illegible]66). Such a large asymmetry in unidirectional fluxes can only point to paracellular solvent drag as the cause. We suggest such drag is driven by electroosmosis along the paracellular route.

**Keywords** Cornea · Endothelium · Fluid transport · Electroosmosis · Solvent drag

## Introduction

For any fluid transporting epithelium, the mechanism by which electrolyte and fluid movements are coupled, as well as the route traversed by the transported fluid, remain contested (Hill et al. 2004; Fischbarg 2010). Local osmosis through membrane water channels has been invoked as the coupling mechanism (Mathias and Wang 2005), but recent evidence that fluid transport can proceed in the absence of membrane water channels (Maclaren et al. 2014), or in the absence of ion transport (hence, no osmosis) (Diecke et al. 2007; Fischbarg 2010; Hong et al. 2014) casts doubts on such explanation. In contradistinction, we have recently proposed that electroosmosis through the paracellular junctions can explain corneal endothelial fluid transport. In those papers (Sanchez et al. 2002; Fischbarg 2003; Rubashkin et al. 2005; Fischbarg and Diecke 2005; Fischbarg et al. 2006; Fischbarg 2010), we presented evidence that fluid movement appears to be driven across the paracellular pathways by an intense electrical current. This current traverses a cellular route from the apical to the basolateral side, and returns to the apical side through the intercellular spaces, thus dragging fluid electro-osmotically along the intercellular junctions.

In the current work, we have therefore tested whether there is solvent drag of solute (Larsen 2002) through the intercellular or paracellular (Sofia Hernandez et al. 1995) route. We chose to use cultured human corneal endothelial cells (HCE cells) (Bednarz et al. 2000), which are known to transport fluid in vitro; the endothelia of other species do it as well, in vivo and in vitro (cultured) (Dikstein and Maurice 1972; Narula et al. 1992). We utilized fluorescein-$Na_2$ (Sigma Chem. Co., St Louis, MO.), which is a well-known extracellular marker (Cvenkel et al. 2015), and is used to determine paracellular permeability (Chang and Karasov 2004).

## Methods

SV-40-transformed Human Corneal Endothelial (HCE) layers (J. Bednarz's line) (Bednarz et al. 2000) were grown to confluence (4–6 days) on permeable membrane inserts

**Electronic supplementary material** The online version of this article (doi:10.1007/s00232-015-9849-y) contains supplementary material, which is available to authorized users.

✉ J. Fischbarg
jf20@columbia.edu

[1] ININCA, Conicet, Marcelo T. de Alvear 2270, CP 1122AAJ Buenos Aires, Argentina

**Table 1** Unidirectional fluorescein fluxes across cultured human endothelium

| n | | Sample Ap to Bas | Corr size destin. comp. 52*samp | Avg Ap to Bas w Corr | Ap-Bas sem | Sample Bas to Ap. | Corr size destin. comp. 30*samp | Corr for ap size equal to basolat 1.73333*col J | Avg Bas to Ap w Corr | B-A sem |
|---|---|---|---|---|---|---|---|---|---|---|
| 1 | Insert 1 | 57,712 | 3,001,033 | 2,994,467 | 6,566 | 61,902 | 1,857,071 | 4,828,384 | 4,960,9[illegible] | 132,520 |
| 2 | | 57,460 | 2,987,901 | | | 65,300 | 1,959,009 | 5,093,423 | | |
| 3 | Insert 2 | 84,798 | 4,409,486 | 4,218,897 | 182,549 | 88,669 | 2,660,081 | 6,916,209 | 6,919,644 | 91,298 |
| 4 | | 84,486 | 4,393,285 | | | 86,708 | 2,601,252 | 6,763,256 | | |
| 5 | | 74,114 | 3,853,919 | | | 90,762 | 2,722,872 | 7,079,467 | | |
| 6 | Insert 3 | 111,107 | 5,777,574 | 5,936,030 | 158,455 | 123,098 | 3,692,942 | 9,601,[illegible] | 9,97[illegible] | 374,115 |
| 7 | | 117,202 | 6,094,485 | | | 132,691 | 3,980,723 | 10,34[illegible],879 | | |
| 8 | Insert 4 | 63,365 | 3,294,957 | 3,234,089 | 51,761 | 67,714 | 2,031,411 | 5,2[illegible],668 | 5,2[illegible]9,814 | 41,661 |
| 9 | | 63,003 | 3,276,174 | | | 69,410 | 2,082,307 | 5,41[illegible]98 | | |
| 10 | | 60,214 | 3,131,135 | | | 67,923 | 2,037,677 | [illegible],297,[illegible] | | |
| | AVGs | 77,346 | | 4,095,870 | | 85,418 | | | 6,786,532 | |
| | S.E.M. | | | | 247,294 | | | | | 409,383 |
| | Δ (Net fl B to A) | 2,690,661 | | | | | | | | |
| | SEM net | 478,277 | | | | | | | | |
| | Net/sem | 5.63 | t(9, 5.63,2) | 0.0003 | | | | | | |
| | Ratio unidirectionals | 1.66 | ± | 0.18 | | | | | | |
| | Size apex (upper c.) | 1.5 ml | | | | | | | | |
| | Size base (lower c.) | 2.6 ml | | | | | | | | |
| | Size base/size apex | 1.7333 | | | | | | | | |

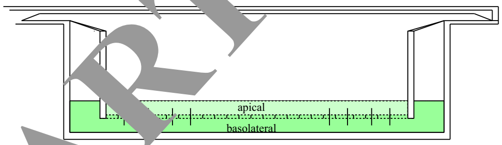


The size of the compartments is asymmetric, but the level of fluid [illegible] same, which nullifies hydrostatic pressure differences. Net Flux appears distinctly, either from the samples, or when compartment size [illegible] is corrected for ($B - A/A - B = 1.66 \pm 0.18$). Net flux is statistically significant ($p = <0.0003$)

RETRACTED ARTICLE

(Transwell Costar #3450). The inserts [illegible]laced in an incubator ($CO_2$ level: 5 %; temperature: 37 [illegible] relative humidity: 90 %). Confluence was ve[illegible]ified visually with a phase-contrast inverted microscope [illegible]Nikon TMS, 200X), and by determining the transendothel[illegible] specific resistance (≈25 Ω $cm^2$ at confluence) u[illegible] an Endohm-24 tissue resistance measurement chamber [illegible] conjunction with a EVOM epithelial volt-o[illegible]eter (both from WPI, Sarasota, FL).

We used Dulbe[illegible]'s [illegible]dified Eagle Medium (DMEM, Gibco BRL) w[illegible] high gl[illegible]cose (4.5 g/l) plus 6 % FBS, penicillin (10[illegible] U/m[illegible] and streptomycin (100 ng/ml), and no phenol red, to avo[illegible] interference with the fluorescein.

$Na_2$-[illegible]escein (Sigma Chem. Co., St Louis, MO.) was diss[illegible]ed di[illegible]tly into the medium (0.15 mg/ml). The fluo[illegible]scei[illegible] labeled medium was added either to the basolatera[illegible] to the apical side of the inserts, while the opposite side c[illegible]ied unlabeled medium. Chamber volumes were 1.5 ml in the upper (apical) compartment and 2.6 ml in the lower (basal) one. After the media were added, the inserts were placed in the incubator for 1 h, and at that point, the entire compartmental volumes were collected. An aliquot (50 μL) of each chamber (mixed with (2.95 mL of saline) was placed in a standard fluorometer vial, and the amount of fluorescein was determined by fluorescence using a Photon Technology International fluorometer (excitation 380 nm; emission 550 nm; 2 nm bwth) in photon counting mode and using Felix software. The samples were read for 60 s.

## Results and Discussion

In four inserts, we were able to determine successfully 20 flux periods of one hour each, 10 in one direction, and 10 in the opposite one (Table 1). We alternated randomly the direction of the flux measured. We determined (Table 1) that the fluorescein unidirectional flux, going from the basolateral towards the apical side (the same direction as fluid transport), was larger than the opposite one (ratio: 1.66 ± 0.18). To avoid a difference in hydrostatic pressure, the level of both apical and basolateral compartments was the same. Hence, the size of the compartments happened to be unequal (basolateral: 2.6 ml; apical: 1.5 ml). Even so, the results were similar whether (a) the apical compartment was assumed equalized in size to the basolateral, (b) or the basolateral compartment was assumed contracted to the size of the apical one (ratio in that case: 1.92) (Tables 1, 2).

**Table 2** As in Table 1, except that the correction for unequal compartment size assumes basolateral size is contracted to the size of the apical pne

| n | | Sample Ap to Bas | Corr size destin. comp. 52*samp/1.7333 | Avg Ap to Bas w Corr | Ap-Bas sem | Sample Bas to Ap. | Corr size destin. comp. 30*samp/0.6759 | Avg Bas to Ap w Corr | B-A sem |
|---|---|---|---|---|---|---|---|---|---|
| 1 | Insert 1 | 57,712 | 1,731,365 | 1,727,577 | 3788 | 61,902 | 3,218,923 | 3,307,2[illegible] | 88,346 |
| 2 | | 57,460 | 1,723,789 | | | 65,300 | 3,395,61[illegible] | | |
| 3 | Insert 2 | 84,798 | 2,543,934 | 2,433,979 | 105,317 | 88,669 | 4,610,8[illegible] | 4,613,0[illegible]6 | 60,866 |
| 4 | | 84,486 | 2,534,588 | | | 86,708 | 4,5[illegible]837 | | |
| 5 | | 74,114 | 2,223,415 | | | 90,762 | [illegible],719,644 | | |
| 6 | Insert 3 | 111,107 | 3,333,216 | 3,424,632 | 91,416 | 123,098 | [illegible]401,100 | 6,650,510 | 249,410 |
| 7 | | 117,202 | 3,516,049 | | | 132,691 | [illegible]9,920 | | |
| 8 | Insert 4 | 63,365 | 1,900,937 | 1,865,820 | 29,862 | 67,714 | 3,52[illegible]12 | 3,554,139 | 27,774 |
| 9 | | 63,003 | 1,890,100 | | | 69,4[illegible]0 | [illegible]609,332 | | |
| 10 | | 60,214 | 1,806,424 | | | 67,[illegible] | 3,[illegible]31,974 | | |
| | **AVGs** | 77,346 | | 2,363,002 | | [illegible]5,41[illegible] | | 4,531,254 | |
| | **S.E.M.** | | | | 142,670 | | | | 272,922 |
| | **Δ (Net fl B to A)** | 2,168,251 | | | | | | | |
| | **Error net** | 307,963 | | | | | | | |
| | **Statistics Ratio** | 7.04 | t(7.04,8,2) | 0.0001 | | | | | |
| | **Unidirectionals** | 1.92 | | | | | | | |
| | Size apex (upper c.) | 1.5 | ml | | | | | | |
| | Size base (lower c.) | 2.6 | ml | | | | | | |
| | Base/ apex | 1.7333 | | | | | | | |
| | Apex/ base | 0.5769 | | | | | | | |

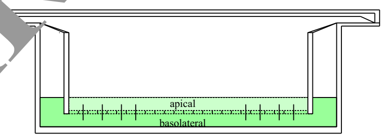


RETRACTED ARTICLE

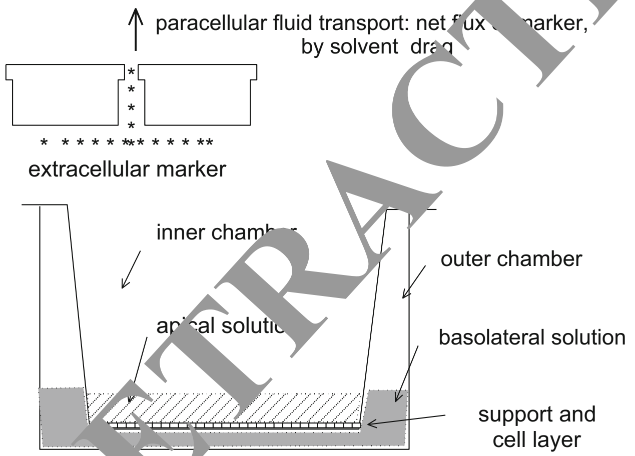


**Fig. [illegible]** *Top* fluorescein marker permeates the paracellular, but not the cell membrane. *Bottom* costar insert placed inside its well, with the endothelial layer grown on *top*. Graph depicts the *outer* and *inner* compartments filled with DMEM solution

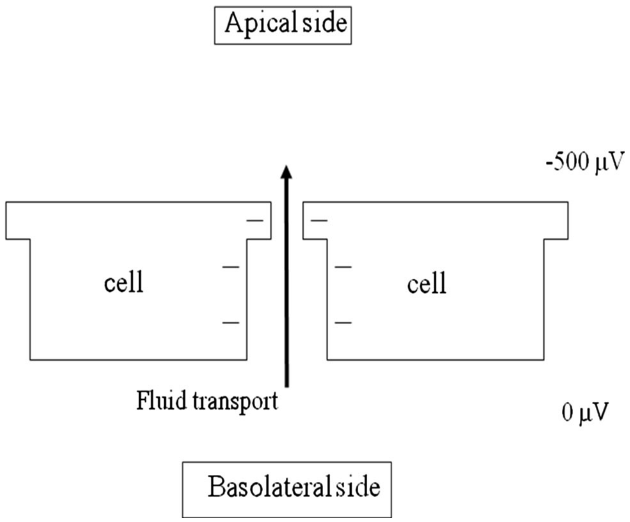


**Fig. 2** Schematic view of two endothelial cells and the intercellular (paracellular) route, depicting fluid transport across such route

From the data in the original paper for this cell line (Aboalchamat et al. 1999), one can calculate an active (basolateral to apical) linear flux of 133 µm/h. Converting into units that include the surface area, this translates into an active fluid flux component (fluid transport equivalent) of 13.3 $\mu L\ h^{-1}\ cm^{-2}$. This is somewhat lower than

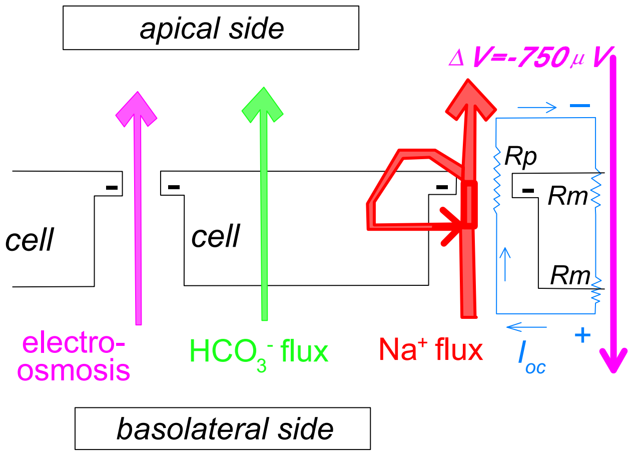


**Fig. 3** Electroosmosis: a schematic description of the transendothelial routes for ionic fluxes, electrical currents, and fluid movements. Note the intense paracellular electro-osmotic current carried by $Na^{+}$ ions. From J Fischbarg Physiol Revs 2011

observed before, but still of the same order of magnitude than seen before in this and other endothelial preparations (for rabbit endothelium, 45–60 μL/(h $cm^2$) (Dikstein and Maurice 1972; Fischbarg and Lim 1974; Narula et al. 1992).

As for the magnitude of the passive leak, equating that rate above (13.3 μL $h^{-1}$ $cm^{-2}$) to the present active flux component, the passive flux (per unit area) becomes 20.25 μL $h^{-1}$ $cm^{-2}$, of the same order than that of the active flux. This makes sense, as the junction is a relative restriction but still is wide enough (≈40 [illegible]) to allow a sizable active flux (fluid transport), and hence it should also allow passive unidirectional fluxes of a similar order.

At the basal end, the intercellular spaces (≈200 Ä wide) are instead quite open and communicating freely with the basal space. Hence, any hypothetical osmotic transfer of fluid from the cell into the intercellular spaces, or any hypothetical hydrostatic pressure buildup in the spaces, would drive the fluid predominantly (5:1) through the open basal end. This direction (apical to basal) is *exactly opposite* to the direction fluid transport moves experimentally. Hence, one is driven to admit that whatever combination one chooses of cellular water channels (aquaporins) and/or classical osmosis fails to explain fluid transport in the direction experimentally observed. Instead, the present findings suggest (once more) the presence of a potent electroosmotic impelling force along the paracellular space and junction, from basolateral towards apical. The accompanying diagrams (Figs. 1, 2, 3) are addressed to summarize this mechanism.

In other epithelia (Hill 1975; Hemlin 1995) and in this one (Lyslo et al. 1985), electroosmosis has been considered before in passing as an explanation of the driving force. We joined this chorus somewhat later, but came to offer weightier evidence for it (this paper, and see refs. in the Introduction). In recent years, reviewers in ocular epithelia (Bonanno 2012; Candia and Alvarez 2008) have also begun guardedly mentioning electroosmosis as a contender for the explanation of fluid transport. One might ask aloud whether in other epithelia fluid transport will be eventually also shown to be due to paracellular electroosmosis, or whether classical osmosis will be somehow revived as an explanation. The accumulation of evidence for electroosmosis, including the present paper, makes such revival somewhat unlikely. In fact, the present idea of electroosmosis could find application in artificial kidneys, which would thus become significantly faster.

**Acknowledgments** Support ([illegible]JF): NIH Grant EY 06178; RPB, Inc; and Argentine Agency for Promotion of Res. & Development, subsidy 0901-2011. We are grateful to Dr. Jürgen Bednarz for his kind gift of cultured human endothelial cells.

**Open Access** This article is distributed under the terms of the Creative Commons Attribution 4.0 International License (http://creativecommons.org/licenses/by/4.0/), which permits unrestricted use, distribution, and reproduction in any medium, provided you give appropriate credit to the original author(s) and the source, provide a link to the Creative Commons license, and indicate if changes were made.

## References


Aboalchamat B, Engelmann K, Bohnke M, Eggli P, Bednarz J (1999) Morphological and functional analysis of immortalized human corneal endothelial cells after transplantation. Exp Eye Res 69:547–553

Bednarz J, Teifel M, Friedl P, Engelmann K (2000) Immortalization of human corneal endothelial cells using electroporation protocol optimized for human corneal endothelial and human retinal pigment epithelial cells. Acta Ophthalmol Scand 78:130–136

Bonanno JA (2012) Molecular mechanisms underlying the corneal endothelial pump. Exp Eye Res 95:2–7

Candia OA, Alvarez LJ (2008) Fluid transport phenomena in ocular epithelia. Prog Retin Eye Res 27:197–212

Chang MH, Karasov WH (2004) Absorption and paracellular visualization of fluorescein, a hydrosoluble probe, in intact house sparrows (*Passer domesticus*). Zoology (Jena) 107:121–133

Cvenkel B, Stunf S, Srebotnik Kirbis I, Strojan Flezar M (2015) Symptoms and signs of ocular surface disease related to topical medication in patients with glaucoma. Clin Ophthalmol 9:625–631

Diecke FP, Ma L, Iserovich P, Fischbarg J (2007) Corneal endothelium transports fluid in the absence of net solute transport. Biochim Biophys Acta 1768:2043–2048

Dikstein S, Maurice DM (1972) The metabolic basis of the fluid pump in the cornea. J Physiol 221:29–41

Fischbarg J (2003) On the mechanism of fluid transport across corneal endothelium and epithelia in general. J Exp Zool A 300:30–40

Fischbarg J (2010) Fluid transport across leaky epithelia: central role of the tight junction, and supporting role of aquaporins. Physiol Rev 90:1271–1290

Fischbarg J, Diecke FP (2005) A mathematical model of electrolyte and fluid transport across corneal endothelium. J Membr Biol 203:41–56

Fischbarg J, Lim JJ (1974) Role of cations, anions and carbonic anhydrase in fluid transport across rabbit corneal endothelium. J Physiol 241:647–675

Fischbarg J, Diecke FP, Iserovich P, Rubashkin A (2006) The role of the tight junction in paracellular fluid transport across corneal endothelium. Electro-osmosis as a driving force. J Membr Biol 210:117–130

Hemlin M (1995) Fluid flow across the jejunal epithelia in vivo elicited by d-c current: effects of mesenteric nerve stimulation. Acta Physiol Scand 155:77–85

Hernandez CS, Gonzalez E, Whittembury G (1995) The paracellular channel for water secretion in the upper segment of the Malpighian tubule of *Rhodnius prolixus*. J Membr Biol 148: 233–242

Hill AE (1975) Solute-solvent coupling in epithelia: an electro-osmotic theory of fluid transfer. Proc R Soc Lond B 190: 115–134

Hill AE, Shachar-Hill B, Shachar-Hill Y (2004) What are aquaporins for? J Membr Biol 197:1–32

Hong JH, Park S, Shcheynikov N, Muallem S (2014) Mechanism and synergism in epithelial fluid and electrolyte secretion. Pflug Arch 466:1487–1499

Larsen EH, Hans H (2002) Using—scientific work: contemporary significance and perspectives. Biochim Biophys Acta 1566:2–15

Lyslo A, Kvernes S, Garlid K, Ratkje SK (1985) Ionic transport across corneal endothelium. Acta Ophthalmol (Copenh) 63:116–125

Maclaren OJ, Sneyd J, Crampin EJ (2014) What do aquaporin knockout studies tell us about fluid transport in epithelia? J Membr Biol 246:297–305

Mathias RT, Wang H (2005) Local osmosis and isotonic transport. J Membr Biol 208:39–53

Narula PM, Xu M, Kuang K, Akiyama R, Fischbarg [illegible] (1992) Fluid transport across cultured bovine corneal endothelial cell monolayers. Am J Physiol 262:C98–C103

Rubashkin A, Iserovich P, Hernandez J, Fischbarg J (2005) Epithelial fluid transport: protruding macromolecules and space charges can bring about electro-osmotic coupling at the tight junctions. J Membr Biol 208:251–263

Sanchez JM, Li Y, Rubashkin A, Iserovich P, Wen Q, Ruberti JW, Smith RW, Rittenband D, [illegible]ang K, Diecke FPJ, Fischbarg J (2002) Evidence [illegible] a central role for electro-osmosis in fluid transport by corneal [illegible]dothelium. J Membr Biol 187:37–50

RETRACTED ARTICLE
